# Unraveling the role of Microglia in triggering High Fat Diet‐Induced Cognitive Decline

**DOI:** 10.1002/alz.090755

**Published:** 2025-01-03

**Authors:** Andreza Fabro de Bem, Priscila Batista Da Rosa, Matheus Scarpatto Rodrigues, Daiane F Engel, Gisela Paola Lazzarino, Jade de Oliveira, David Engblom

**Affiliations:** ^1^ Universidade de Brasília, Brasília Brazil; ^2^ Linkoping University, Linkoping Sweden; ^3^ Universidade Federal do Rio Grande do Sul, Porto Alegre Brazil; ^4^ Linköping University, Linköping Sweden

## Abstract

**Background:**

Excessive dietary fat is not only a risk factor for metabolic disorders but also for premature cognitive decline and Alzheimer’s disease. Recent findings from our study revealed that even a few days of a high‐fat diet (HFD) are sufficient to disrupt hippocampal bioenergetics, activate microglia, and induce cognitive decline in mice. We hypothesize that microglia, rather than merely responding to diet‐induced damage, play a critical role in disrupting synaptic homeostasis.

**Method:**

To investigate this hypothesis, we first assessed the effects of a HFD over time on cognitive impairment and hippocampal microglia activation in C57Bl6J male mice. Subsequently, we employed a chemogenetic approach using Designer Receptors Exclusively Activated by Designer Drugs (DREADD) to conditionally inhibit microglia and evaluate the metabolic and behavioral outcomes induced by HFD.

**Result:**

Remarkably, only 10 days of HFD led to changes in hippocampal‐dependent behavioral tasks, such as the novel object recognition test, in 3‐month‐old male C57Bl6J mice, which persisted for 4 and 8 weeks. Sholl analysis and stereology confirmed morphological alterations and reactivation of microglia in the hippocampal CA1 region after 10 days of HFD, while metabolic parameters (adiposity and weight gain) were increased only after 4 and 8 weeks of HFD in C57Bl6J mice. Interestingly, sub‐chronic chemogenetic microglial inhibition (by DREADD) prevented memory impairment, evaluated by novel object recognition and object reallocation tests, in mice submitted to HDF for 7 days.

**Conclusion:**

Together our data support the notion that microglia play a critical role in triggering mechanisms of hippocampal damage induced by HFD.

